# Combination of microbiome analysis and serodiagnostics to assess the risk of pathogen transmission by ticks to humans and animals in central Germany

**DOI:** 10.1186/s13071-018-3240-7

**Published:** 2019-01-07

**Authors:** Yvonne Regier, Kassandra Komma, Markus Weigel, Peter Kraiczy, Arttu Laisi, Arto T. Pulliainen, Torsten Hain, Volkhard A. J. Kempf

**Affiliations:** 1 University Hospital, Goethe-University, Institute for Medical Microbiology and Infection Control, Frankfurt am Main, Germany; 2 Institute of Medical Microbiology, Justus-Liebig University, Giessen, Germany; 3 Institute of Biomedicine, Research Center for Cancer, Infections and Immunity, University of Turku, Turku, Finland; 4 German Centre for Infection Research (DZIF), partner site Giessen-Marburg-Langen, Giessen, Germany

**Keywords:** *Bartonella*, Microbiome, Tick, Dog, Roe deer, Nanopore, Illumina, One health

## Abstract

**Background:**

Arthropod-borne diseases remain a major health-threat for humans and animals worldwide. To estimate the distribution of pathogenic agents and especially *Bartonella* spp., we conducted tick microbiome analysis and determination of the infection status of wild animals, pets and pet owners in the state of Hesse, Germany.

**Results:**

In total, 189 engorged ticks collected from 163 animals were tested. Selected ticks were analyzed by next generation sequencing (NGS) and confirmatory PCRs, blood specimens of 48 wild animals were analyzed by PCR to confirm pathogen presence and sera of 54 dogs, one cat and 11 dog owners were analyzed by serology. *Bartonella* spp. were detected in 9.5% of all ticks and in the blood of 17 roe deer. Further data reveal the presence of the human and animal pathogenic species of genera in the family *Spirochaetaceae* (including *Borrelia miyamotoi* and *Borrelia garinii*), *Bartonella* spp. (mainly *Bartonella schoenbuchensis*), *Rickettsia helvetica*, *Francisella tularensis* and *Anaplasma phagocytophilum* in ticks. Co-infections with species of several genera were detected in nine ticks. One dog and five dog owners were seropositive for anti-*Bartonella henselae-*antibodies and one dog had antibodies against *Rickettsia conorii.*

**Conclusions:**

This study provides a snapshot of pathogens circulating in ticks in central Germany. A broad range of tick-borne pathogens are present in ticks, and especially in wild animals, with possible implications for animal and human health. However, a low incidence of *Bartonella* spp., especially *Bartonella henselae*, was detected. The high number of various detected pathogens suggests that ticks might serve as an excellent sentinel to detect and monitor zoonotic human pathogens.

## Background

Globalization and climate change both contribute to the spread of infectious diseases often transmitted by insects and arthropod vectors. The monitoring and control of vector-borne diseases is an important task in public health, consolidating medical, veterinary and environmental research to realize potential health threats and to avert and reduce them [1].

Ticks are distributed worldwide and are of special interest in infection epidemiology research as these vectors are able to transmit a broad variety of infectious agents to humans and animals. Hard ticks usually feed three times during their life-cycle and pathogens can be acquired or transmitted during every blood meal. Furthermore, pathogens are transmitted transstadially from larvae to nymphs or from nymphs to adults, or vertically from mother ticks to eggs [2]. For the last two decades, pathogen-harboring-ticks were mainly analyzed by PCR-based applications, e.g. for *Borrelia* spp. and *Bartonella* spp. [3–5]. However, these analyses were limited naturally in their spectrum of detectable pathogens. With the availability of next generation sequencing (NGS) technologies, several studies have analyzed the microbiome of ticks, and besides endosymbionts, a large variety of bacterial pathogen DNA has been found (e.g. *Francisella* spp., *Rickettsia* spp., *Anaplasma* spp., *Bartonella* spp. and *Borrelia* spp.) without the need to select particular tests in advance [6–12].

*Bartonella* spp. are typical examples for vector-borne pathogens. These Gram-negative, facultative intracellular bacteria cause long-lasting intraerythrocytic infections in their respective reservoir hosts and are usually transmitted by blood sucking arthropods [13–15]. For example, rodents and bats serve as primary reservoirs for various *Bartonella* spp., including species with medical relevance for humans [16, 17]. Today, *Bartonella henselae* is the most common pathogenic representative of the genus *Bartonella*. Its reservoir host is the cat from which it is transmitted to humans (causing cat-scratch disease and other diseases) and dogs (causing endocarditis, fever of unknown origin and peliosis hepatis) [13, 18–20]. *Bartonella schoenbuchensis* was isolated first from the blood of wild roe deer in 1999 [21] and it turned out that several ruminant species serve as a reservoir hosts for this particular pathogen [22–29]. In animal reservoir hosts, asymptomatic infections with *Bartonella* spp. are common, although their pathogenicity remains unclear [30, 31]. *Bartonella schoenbuchensis* has been suggested to cause deer ked dermatitis in humans [30] and was isolated from a patient with a history of tick bites who suffered from fatigue, muscle pain and fever [32]. Currently, at least 37 *Bartonella* spp. are known to infect humans and animals [33].

In ticks, the prevalence of *B. henselae* DNA has been demonstrated to be up to ~40% [3] and, although controversially discussed [34], ticks are suspected to transmit *Bartonella* spp. [35]. The vector-competence of ticks has been confirmed in a murine infection model [36] and by using an artificial feeding system [37]. Several studies have shown that various tick species harbor several pathogenic bacteria alongside with *Bartonella* spp. [5, 38, 39], leading to a potential risk of co-infections in humans and animals. Two studies have reported co-infections with *Borrelia burgdorferi* [not specified, respectively *B. burgdorferi* (*sensu lato*)] and *B. henselae* in humans [40, 41]. As co-infections can result in more severe and irregular courses of disease, studies of the microbiome are a crucial prerequisite to estimate the health threat for humans and animals arising from tick bites and allow broader insights in the epidemiology of tick-borne pathogens.

We investigated the presence of *Bartonella* spp. and other pathogens in feeding ticks and blood of pets and wild animals in central Germany (federal state of Hesse) by combining NGS and conventional PCRs for pathogen detection. Moreover, we attempted to detect pathogen-specific antibodies in the serum of pets and their owners.

## Methods

### Sample collection

**Fig. 1:**
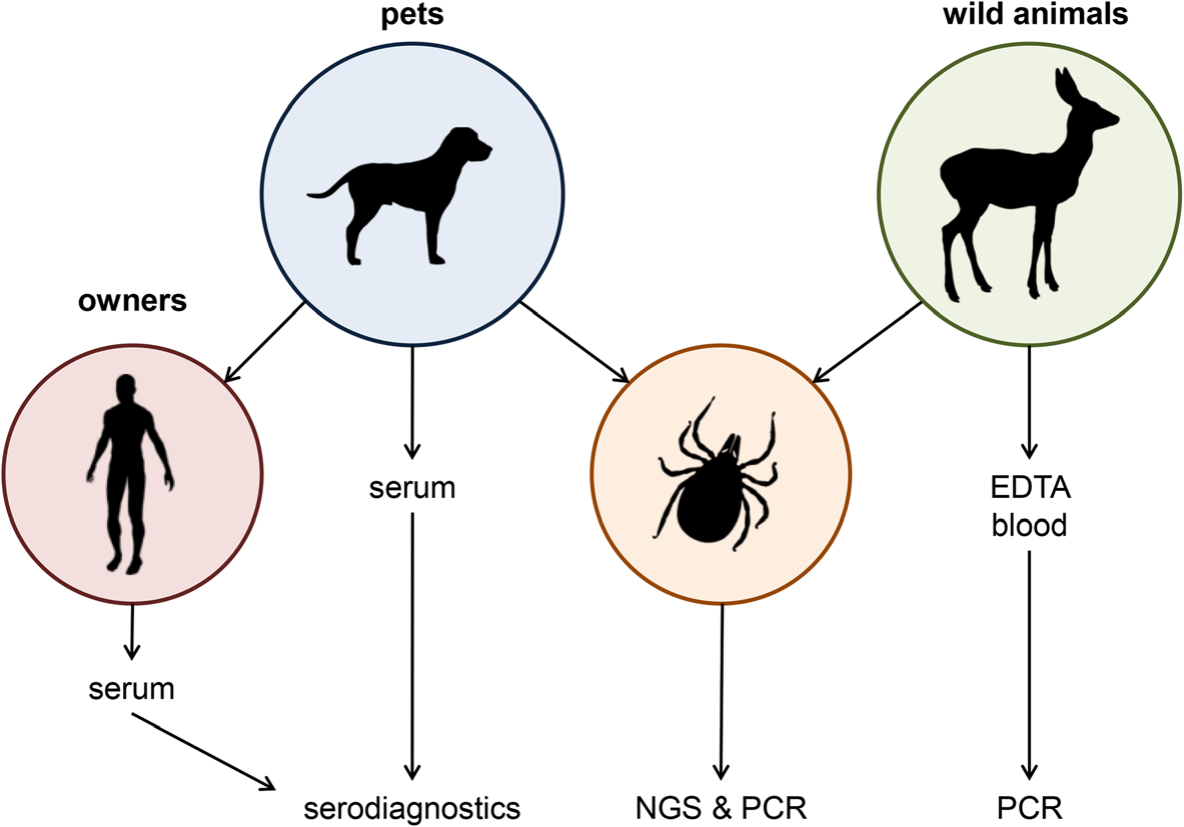
Workflow of all samples. DNA of ticks and animal blood samples was extracted and PCRs for *Bartonella*-specific genes (*16S* rDNA, 16S-23S ITS) were conducted (with subsequent Sanger-sequencing of the amplicons). *16S* rDNA metagenomics was used for determination of the tick microbiome (confirmed by specific PCRs) revealing the presence of further pathogens. Serum of pets and, if available, of pet owners was analyzed for serological infection markers (antibodies) known to indicate previous infections in regard to the molecular findings from ticks

Ticks and serum from pets were collected by veterinarians located in the state of Hesse, Germany, and tick and blood samples from wild animals were collected by hunters directly after shooting and by employees of the Landesbetrieb Hessisches Landeslabor, Gießen, Germany. All locations are given in Table 1. Blood was collected in EDTA- and serum-tubes and ticks were stored in sterile, DNA-free vials (Eppendorf, Hamburg, Germany) containing 70% DNA-free ethanol. Human blood samples were taken by general practitioners or in the outpatient clinics of the Institute for Medical Microbiology and Infection Control, Frankfurt am Main, Germany. The workflow of all samples is shown in Fig. 1.

**Table 1:** Geographical coordinates of hunting sites, veterinary practices and state health authorities in Germany where samples were taken

Animal species (*n*)	Location	Geographical coordinates
Hunting sites		
Boar (*n* = 1)	Urban forest, Frankfurt am Main	50°04'19.8"N, 8°40'52.2"E
Roe deer (*n* = 9); boar (*n* = 3); raccoon (*n* = 1)	Vogelsberg, Schotten	50°31'00.8"N, 9°14'30.1"E
Roe deer (*n* = 3)	Hainchen	50°51'15.912"N, 8°12'57.51"E
Roe deer (*n* = 1)	Koenigstein	50°10'43.464"N, 8°28'18.876"E
Roe deer (*n* = 1)	Altenhain, Taunus	50°9'22.428"N, 8°28'14.048"E
Roe deer (*n* = 1)	Hofheim-Wallau	50°3'43.186"N, 8°22'20.345"E
Roe deer (*n* = 7)	Biedenkopf	50°54'24.35"N, 8°32'9.55"E
Roe deer (*n* = 6); boar (*n* = 1)	Neu-Anspach, Gruenwiesenweiher	50°19'33.8"N, 8°29'38.4"E
Roe deer (*n* = 1)	Buedingen	50°17'10.667"N, 9°6'40.982"E
Roe deer (*n* = 3)	Biblis / Wattenheim	49°41'28.0"N, 8°24'24.9"E
Roe deer (*n* = 2)	Gedern	50°26'54.0"N, 9°14'21.9"E
Veterinary practices and government agencies		
Dog (*n* = 19)	Oberursel	50°12'16.7"N, 8°35'40.3"E
Dog (*n* = 4)	Hattersheim	50°04'11.2"N, 8°28'22.1"E
Dog (*n* = 1)	Bad Vilbel	50°11'13.2"N, 8°44'24.5"E
Dog (*n* = 1); cat (*n* = 1)	Offenbach	50°06'14.3"N, 8°45'19.9"E
Dog (*n* = 2)	Bad Homburg	50°13'11.8"N, 8°38'46.4"E
Dog (*n* = 16)	Frankfurt am Main	50°07'00.3"N, 8°38'35.7"E
Dog (*n* = 2)	Frankfurt am Main	50°10'50.5"N, 8°39'37.9"E
Dog (*n* = 1)	Frankfurt am Main	50°05'11.2"N, 8°35'05.2"E
Dog (*n* = 2)	Hofheim	50°03'52.7"N, 8°23'15.7"E
Dog (*n* = 3)	Moerfelden-Walldorf	49°59'43.5"N, 8°34'34.1"E
Dog (*n* = 1)	Dreieich	50°01'07.8"N, 8°40'23.8"E
Dog (*n* = 1)	Dreieich	50°01'14.7"N, 8°41'12.3"E
Dog (*n* = 1)	Frankfurt am Main	50°08'49.0"N, 8°40'00.1"E
Roe deer (*n* = 3); boar (*n* = 1); red fox (*n* = 1); wisent (*n* = 1)	Landesbetrieb Hess, Landeslabor Gießen	50°34'03.2"N, 8°39'45.2"E
Roe deer (*n* = 2)	Forestry district, Koenigstein	50°10'43.464"N, 8°28' 18.876"E

### DNA-extraction from ticks and whole-blood

The laboratories of the Institute for Medical Microbiology and Infection Control at the University Hospital of the Goethe University in Frankfurt (Germany) undergo a strict and externally reviewed quality control management with all required positive and negative controls (laboratory accreditation according to ISO 15189:2014 standards; certificate number D-ML-13102-01-00, valid through January 25th, 2021) and are appointed as National Consiliary Laboratories for *Bartonella* by the Robert Koch Institute, Berlin, Germany. There was no increase of *Bartonella-*positive cases during this study; therefore, the possibility of DNA contamination from non-study sources is highly unlikely.

DNA extraction from ticks was conducted as previously described [42]. All ticks were individually removed from tubes with sterile forceps, identified using standard taxonomic keys [43] and washed twice in sterile ethanol and once in sterile water to avoid DNA contamination by environmental microorganisms present on the cuticle of the ticks. Each tick was treated individually to prevent DNA cross-contamination. DNA was extracted by using the QIAamp DNA Mini kit (Qiagen, Hilden, Germany) according to manufacturer’s instructions and eluted in 200 μl elution buffer. Grinding was conducted with disposable sterile mortars and pestles. The extraction procedure was strictly controlled using specific pathogen-free ticks (Insect Services, Berlin, Germany) in which *Bartonella* spp. were never detected (data not shown). DNA from whole-blood was extracted using the DNeasy Blood and Tissue Kit (Qiagen) and eluted in 200 μl of elution buffer as recommended by the manufacturer.

### PCR-detection of pathogen-specific genes by PCR

In this study, we focused on the detection of *Bartonella* spp. in addition to other pathogens. Therefore, all ticks and EDTA-blood were analyzed for *Bartonella* spp. DNA using two different PCR methods [42]: *16S* ribosomal DNA (rDNA) nested PCR [3, 44] using the Taq DNA Polymerase-Kit (Invitrogen, Schwerte, Germany) and 16S-23S rDNA internal transcribed spacer (ITS) region PCR [45] using the Platinum Taq Polymerase-Kit (Invitrogen). Positive (*B. henselae* Houston ATCC 49882, 1 ng) and negative (water) intra-assay controls were always included. DNA was amplified in a Biometra T3000 thermocycler (Biometra, Goettingen, Germany). Products were separated on agarose gels, stained with ethidium bromide and visualized under UV light. PCR products were sequenced by a commercial provider (GATC, Konstanz, Germany), analyzed using the Chromas software (Technelysium, v.2.6, South Brisbane, Australia) and compared to *Bartonella* spp. strains deposited in the NCBI databank using the BLAST online tool for species level identification.

Whenever potentially pathogenic bacterial species were detected by NGS in the microbiome analysis (see below), subsequent PCRs for species determination were conducted from ticks and from EDTA-blood of the animals, if available. Primers for the detection of the *Bartonella* spp. *16S-*rDNA nested PCR and the *Bartonella* spp. 16S-23S rDNA ITS region PCR, the *Rickettsia* spp. 23S-5S ITS region [46], the *Coxiella burnetii* IS1111 region [47], *16S* rDNA of *Anaplasma* spp. [48], the *Leptospira* spp. *LipL32* gene [49] and *Borrelia* spp. *16S* rDNA [50] are given in Table 2. PCRs were conducted employing the standard protocol for the Platinum Taq Polymerase Kit and all PCR-products were sequenced and analyzed. Detection of the *ospA* gene of *Borrelia* spp. in ticks and of *Francisella tularensis* were conducted using LightMix Kits (TIB MOLBIOL, Berlin, Germany) according to the manufacturer’s instructions. Positive controls (each containing 1 ng of DNA) were the following: *Rickettsia helvetica* (friendly gift of Dr. Dobler, München, Germany), *Coxiella burnetii* (German laboratory quality assessment trials, INSTAND e.v., Düsseldorf, Germany), *Anaplasma phagocytophilum* Webster (friendly gift of Prof. von Loewenich, Mainz, Germany), *Leptospira interrogans* (German Federal Institute for Risk Assessment, Berlin, Germany) and *Borrelia miyamotoi* HT31 (CDC, Fort Collins, USA).

**Table 2:** Targets, primers and amplicon size for the PCR-testing from ticks and EDTA-blood

Target sequence	Designation	Sequence (5'-3')	Amplicon length (bp)	Reference
*Bartonella* spp. *16S* rDNA, 1st round	A-proteo	AGAGTTTGATC(AC)TGGCTCAGA	1210	[44]
	r-Alpha-sh	GTAGCACGTGTGTAGCCCA		
*Bartonella* spp. *16S* rDNA, 2nd round	Bart	CACTCTTTTAGAGTGAGCGGCAA	990	[44]
	r-BH	CCCCCTAGAGTGCCCAACCA		
*Bartonella* 16S-23S ITS region	325s	CTTCAGATGATGATCCCAAGCCTTCTGGCG	various *Bartonella* spp., ~500 bp	[45]
	1100as	GAACCGACGACCCCCTGCTTGCAAAGCA		
*Bartonella* spp. rpoB	prAPT0244	GATGTGCATCCTACGCATTATGG	406	[51]
	prAPT0245	AATGGTGCCTCAGCACGTATAAG		
*Anaplasma* spp. *16S* rDNA 1st round	ge3A	CACATGCAAGTCGAACGGATTATTC	932	[48]
	ge10r	TTCCGTTAAGAAGGATCTAATCTCC		
*Anaplasma* spp. *16S* rDNA 2nd round	ge9f	AACGGATTATTCTTTATAGCTTGCT	546	[48]
	ge2	GGCAGTATTAAAAGCAGCTCCAGG		
*C. burnetii* IS1111	CB_S4k	GAAACGGGTGTTGAATTGTTTG	290	[47]
	CB_A2k	ATCACCAATCGCTTCGTCCCGGT		
*Rickettsia* spp. 23S-5S ITS region	23S for	GATAGGTCGGGTGTGGAAGCAC	various *Rickettsia* spp., ~500 bp	[46]
	23S rev	GGGATGGGATCGTGTGTTTCAC		
*Leptospira* spp. LipL32	LipL32-270F	CGCTGAAATGGGAGTTCGTATGATT	423	[49]
	LipL32-692R	CCAACAGATGCAACGAAAGATCCTTT		
*Borrelia* spp. *16S* rDNA	16S FW	GGCTTAGAACTAACGCTGGCAGTGC	552	[50]
	16S RV	CCCTTTACGCCCAATAATCCCGA		

### Differentiation of ruminant-associated *Bartonella* spp. by *rpoB*-PCR

**Fig. 2:**
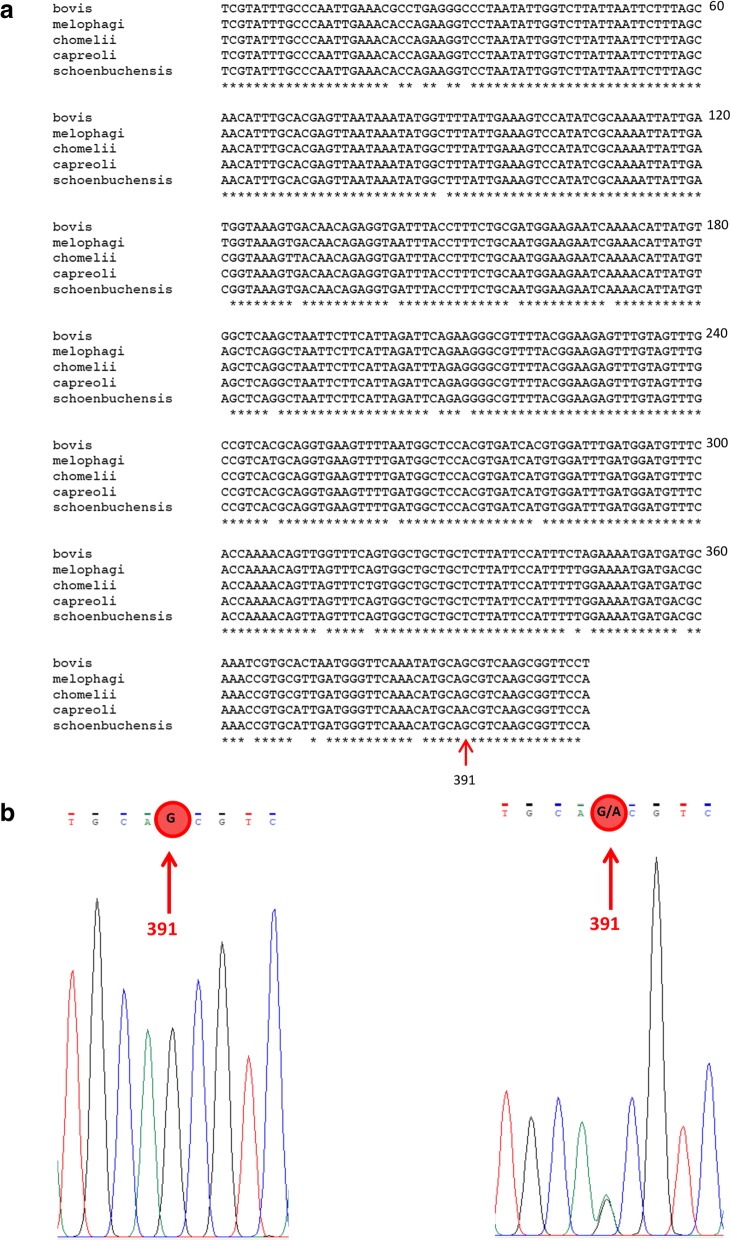
Discrimination of ruminant-associated *Bartonella* spp. by SNP-analysis. **a** Alignment of *B. bovis*, *B. melophagi*, *B. chomelii*, *B. capreoli* and *B. schoenbuchensis.* Discriminatory nucleotide positions are on 27 positions*.* **b** Left: unibacterial *B. schoenbuchensis* infection (sequence at the discriminatory nucleotide …TGCA**G**CGTC…); right: *B. schoenbuchensis* and *B. capreoli*-co-infection (sequence at the discriminatory nucleotide …TGCA**G/A**CGTC…)

To increase the species-discriminatory power of the rDNA-sequences within the *Bartonella* ruminant-lineage, a PCR protocol specific for a 406 bp internal fragment of the *rpoB* gene (β-subunit of the bacterial RNA polymerase) was performed [51]. *Bartonella rpoB* DNA was amplified by using 5 μl of starting material. Positive (*B. henselae* Houston ATCC 49882, 0.5 ng) and a negative (water) controls were always included. Primers are given in Table 2. PCR products were sequenced (GATC) and analyzed using Chromas software. To analyze the *Bartonella* species-discriminatory nucleotide polymorphism of the 403 bp *rpoB* fragment, the obtained sequences were aligned to sequences of *Bartonella schoenbuchensis* (type strain R1, AY167409.1), *B. capreoli* (type strain IBS193, AB290188.1), *Bartonella chomelii* (type strain A828, AB290189.1), *B. bovis* (type strain 91-4, AY166581.1) and *Bartonella melophagi* (type strain K-2C, EF605288.1) using Clone Manager Professional Suite 8 (Scientific and Educational Software, Denver, USA). As shown in Fig. 2, the 406 bp *rpoB* fragment allows species discrimination of *B. chomelii*, *B. bovis* and *B. melophagi* based on multiple nucleotide positions, as well as species discrimination of *B. schoenbuchensis* and *B. capreoli* based on a single nucleotide position (position 391). Co-infections with *B. schoenbuchensis* and *B. capreoli* were detected by analyzing double peaks at the discriminatory nucleotides.

### Microbiome analysis of ticks using next generation sequencing by Illumina technology

The V4 region of the *16S* rRNA gene of each tick was amplified using previously described primers [52]. Amplification was done using the Platinum SuperFi PCR Master Mix (Thermo Fisher Scientifc, Carlsbad, USA). Each PCR reaction was performed in a total of 25 μl of reaction solution comprising 2× SuperFi PCR Master Mix, 1.25 μl of 10 pmol forward and reverse primers and a maximum of 10 μl of DNA per reaction. After mixing the solutions, the following thermocycler conditions were run: 98 °C for 2 min; 25 cycles at 98 °C for 10 s, 55 °C for 10 s and 72 °C for 30 s; and a final extension step at 72 °C for 5 min. The size of PCR products was confirmed on a 2% agarose gel and purified using AMPure XP DNA beads (Beckman Coulter, Brea, USA). The index and adapter ligation PCR was done using a Nextera XT Index Kit v2 Set A and B (Illumina, San Diego, USA) and performed according to the manufacturer’s protocol. PCR conditions were set as follows: 95 °C for 3 min; 8 cycles at 95 °C for 30 s, 55 °C for 30 s and 72 °C for 30 s; and a final extension step at 72 °C for 5 min. Quality and quantity control of purified PCR products were done using a Qubit 2.0 Fluorometer (Thermo Fisher Scientific) and a 2100 Bioanalyzer (Agilent Technologies, Santa Clara, USA). All samples were diluted to the same molarity, pooled together, spiked with an internal control (15% PhiX) and paired-end sequenced on the MiSeq Illumina platform using a flow cell with V2 chemistry (500 cycles). Negative controls (kit and PCR controls) were performed using pure water and elution buffer. In addition, microbial mock communities (Zymo Research, Freiburg, Germany) were run alongside as a standard and as quality control for determining contamination bias of DNA extraction. To ensure the best quality sequencing results, the *16S* rDNA nested PCR and the 16S-23S-rDNA-ITS region PCR were always run in parallel to the NGS were compared. Furthermore, whenever human pathogens were detected by NGS, all positive results were confirmed by conventional PCR methods (see above).

### *16S* full length rRNA gene sequencing using Nanopore

In brief, the entire *16S* rRNA gene (~ 1.5kb) of selected samples was amplified using the native *16S* Barcoding Kit SQK- RAB204 (Nanopore Technologies, Oxford, England). Library preparation and sequencing were done following the manufacturer’s instructions. Each PCR reaction was performed in a total of 50 μl of reaction solution comrpising 14 μl of nuclease-free water, 10 ng of input DNA, 1 μl of barcode and 25 μl of LongAmp Taq 2X Mastermix (New England Biolabs, Frankfurt, Germany). The following PCR protocol was performed: 95 °C for 1 min; 25 cycles at 95 °C for 20 s, 55 °C for 30 s and 65 °C for 2 min, and a final extension step at 72 °C for 5 min. PCR products were purified using 30 μl of AMPure XP DNA beads (Beckman Coulter). Samples were eluted in 10 μl of 10 mM Tris-HCl pH 8.0 with 50 mM NaCl. Quantification of the libraries was performed using a Qubit 2.0 Fluorometer (Thermo Fisher Scientific); subsequently they were pooled together, prepared for loading and eventually sequenced on a R9.4.1 FLO-MIN106 flowcell.

### Bioinformatic microbiome analysis workflow

MiSeq Software v.2.6 was used to split the sequences by barcode and to generate the *fastq* files. The microbiome analysis was done following the MiSeq standard operation procedures [53] using Mothur (v.1.36.1) [54]. Qiime (v.1.9.1) was used for the alpha-diversity calculation and the taxa summary plots [55].

**Fig. 3:**
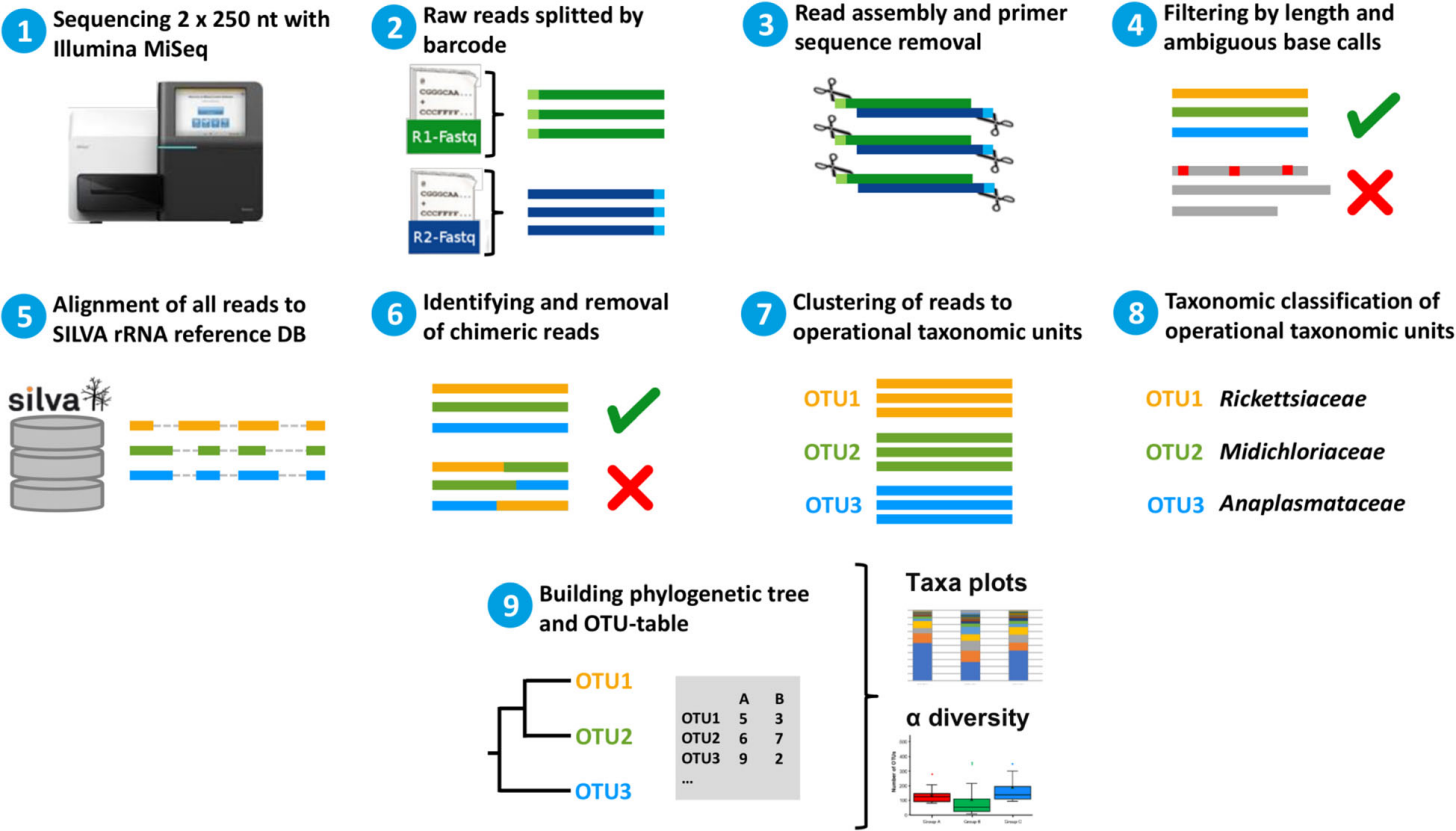
Schematic overview of microbiome bioinformatic analysis workflow. The hypervariable V4 region of *16S* rDNA from tick samples was sequenced and split by barcode with Illumina MiSeq. Resulting paired-end reads were joined and the primer region was removed. Reads were filtered by amplicon length and aligned to SILVA as the reference database. After removal of chimeras, reads were clustered into operational taxonomic units (OTU) and taxonomically classified. Finally, an OTU-table was created and results were visualized

The paired-end reads were joined and the primer sequences were removed. We filtered for the expected amplicon length and removed reads with ambiguous base calls or with homopolymers longer than eight nucleotides. Duplicate sequences were merged. The unique reads were aligned against the SILVA-bases bacterial reference alignment [56]. Nucleotides outside the expected alignment region were trimmed. Reads with a difference of two nucleotides were merged during pre-clustering. Chimeric reads were removed using the Mothur implementation of the uchime algorithm. After chimera removal, taxonomy was assigned and non-bacterial reads were removed. OTUs were created using the cluster split method of Mothur. After clustering, we reassigned the taxonomy to the OTUs. In preparation for the analysis with Qiime, a phylogenetic tree and an OTU table in biom format was created. Alpha-diversity analysis and the taxa summary plots were created using the Qiime core diversity analysis script. The workflow is summarized in Fig. 3. *16S* full length rRNA gene sequencing data were analyzed applying the EPI2ME platform with FASTQ *16S* (v.3.0.0) from Oxford Nanopore Technologies.

### Serodiagnostics of pets and pet owners

All serum samples were screened for the presence of anti-*B. henselae*-antibodies by indirect immunofluorescence assay (IIFA) using a *B. henselae/B. quintana* (IgG) kit (Euroimmun, Luebeck, Germany) according to the manufacturer’s instructions with slight modifications [42]. Pet sera were tested with a 1:100 dilution of Alexa Fluor 488-conjugated AffiniPure Goat Anti-Dog/Cat IgG (Jackson ImmunoResearch laboratories, West Grove, USA) as secondary antibodies. Serum dilutions from 1:20 to 1:2560 were screened for *Bartonella* dog/cat IgG antibodies. Signals were evaluated as positive when specific fluorescence was detected at a titer of ≥ 1:64 [57] for animals and > 1:80 for humans.

Whenever potentially pathogenic genera were found in ticks by NGS or PCR, the corresponding animal sera and, if available, sera of the respective pet owners were analyzed for antibodies against the most common pathogenic bacterial species of these genera. Animal sera were sent to the veterinarian diagnostic laboratory Laboklin (Bad Kissingen, Germany) for the detection of pet antibodies against *Anaplasma phagocytophilum* (ELISA; cut-off: ≥ 8 TE), *Rickettsia conorii* (IIFA) (cut-off: ≥ 1:256), *R. rickettsii* (IIFA; cut-off: IgG ≥ 1:256), *Borrelia* spp. (ELISA; cut-off: ≥ 8 units), *Leptospira* spp. (microagglutination test; cut-off: ≥ 1:400), *Coxiella burnetii* (IIFA; cut-off: ≥ 1:1:20) and *Francisella tularensis* (qualitative serum slow agglutination test).

Serodiagnostics of human sera was performed in the diagnostic laboratories of the Institute for Medical Microbiology and Infection Control at the University Hospital of the Goethe University in Frankfurt am Main (Germany) under fully certified conditions (ISO 15189:2014, certificate number D-ML-13102-01-00, valid through January 25th, 2021). The method used for the detection of antibodies against *Anaplasma phagocytophilum* was IIFA (*A. phagocytophilum* IFA IgG/IgM (Focus Diagnostics, Cypress, CA, USA; cut-off: IgG ≥ 1:64, IgM ≥ 1:20), for *Rickettsia typhi* and *Rickettsia rickettsii* it was IIFA (*Rickettsia* IFA IgG/IgM Focus Diagnostics; cut-off: ≥ 1:64), for *B. burgdorferi* (*sensu lato*) it was ELISA (Enzygnost® Borreliosis/IgM/Lyme link VlsE/ IgG, Siemens, Marburg, Germany; cut-off: ≥ 7 U/ml), for *Leptospira* spp. it was ELISA (*Leptospira* IgG/IgM Serion ELISA classic, Serion, Wuerzburg, Germany; cut-off: IgG ≥ 10 U/ml, IgM ≥15 U/ml) and for *Coxiella burnetii* it was IIFA (*C. burnetii* IgG/IgM IFA Kit, Fuller Laboratories, Fullerton, CA, USA; cut-off ≥ 1:16). Testing for anti*-Francisella tularensis* antibodies was conducted at the Institut für Mikrobiologie der Bundeswehr, München, Germany by ELISA (qualitative cut-off OD 0.25).

## Results

### Sample collection

**Fig. 4:**
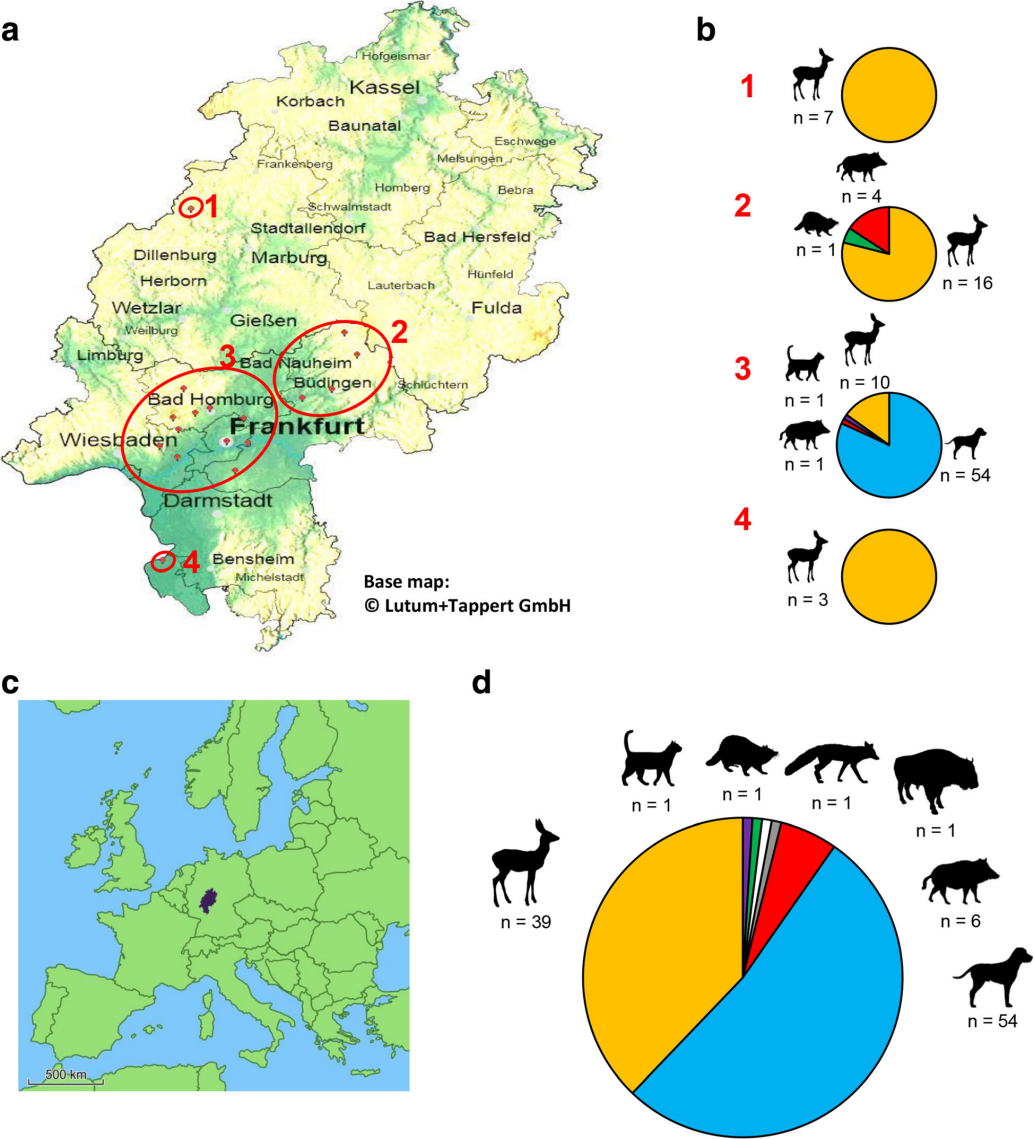
**a** Geographical map of the federal state of Hesse (Germany) displaying the locations of feeding-tick collections. The red marks represent the locations where the ticks were collected. From top to bottom, numbers in red: 1, North Hesse; 2, Mid-west Hesse; 3, Greater metropolitan area Frankfurt am Main; 4, South Hesse. The base map was generated using EasyMap 11.0 © Lutum+Tappert DV-Beratung GmbH. **b** Distribution of sampled ticks and their hosts in relation to their location. From top to bottom: 1, North Hesse; 2, Mid-west Hesse; 3; Greater metropolitan area Frankfurt am Main; 4, South Hesse. **c** Map of Europe with exact location of Hesse tagged. **d** Fractions of all animals examined in this study: dogs, roe deer, cat, raccoon, fox, wisent and boars

From March to October 2017, 189 engorged ticks from 103 animals were collected at four collection sites in the federal state of Hesse, Germany shown in Fig. 4a. Pet samples comprised samples from 54 dogs (54 dog sera, 84 ticks, 17 whole-blood samples) and one cat (serum, whole-blood and one tick). Pet owner samples comprised 11 sera obtained from dog-owners. The samples of wild animals comprised samples from 39 roe deer (*Capreolus capreolus*; 39 whole-blood samples and 95 ticks), 6 boar (*Sus scrofa*; 6 whole-blood samples and 6 ticks), 1 wisent (*Bos bonasus*; 1 whole-blood sample and 1 tick), 1 raccoon (*Procyon lotor*; 1 whole-blood sample and 1 tick) and one red fox (*Vulpes vulpes*; 1 whole-blood sample and 1 tick). The number of sampled animals is summarized in Fig. 4d. Ticks were mostly female adult *Ixodes ricinus* ticks (identified by standard taxonomic keys). Among the 189 ticks, two were nymphs and 6 were male adults. Two tick samples (1 dog and 1 cat) were identified as *Rhipicephalus sanguineus*.

### PCR detection of *Bartonella* spp.

In addition to the detection of multiple pathogens by microbiome analysis, we focused on the detection of *Bartonella* spp. since there is a broad spectrum of *Bartonella* spp. that can lead to severe infections in humans and animals [33]. *Bartonella* spp. were detected in ticks and full blood of roe deer. DNA of two different *Bartonella* spp. was found in roe deer blood: *B. schoenbuchensis* in 10 (25.6%) animals, *B. capreoli* in 4 (10.3%) animals and a co-infection of both *Bartonella* spp. was detected in the blood of 3 (7.7%) roe deer. Among the roe deer with blood positive for *Bartonella* spp., nine did not have *Bartonella* spp.-positive ticks. DNA of *Bartonella* spp. was found in 9.5% of all ticks. *Bartonella schoenbuchensis* DNA was detected in 14 ticks (7.4% of all ticks) from 10 roe deer, *B. capreoli* DNA was found in one tick (0.5% of all ticks) and *B. henselae* DNA was detected in three ticks (1.6% of all ticks) of three roe deer. Moreover, *Bartonella* spp. DNA was found in ticks of three animals whose blood contained no *Bartonella* spp. DNA.

### Microbiome analysis of collected tick samples throughout the state of Hesse

It is widely known that ticks act as vectors for various pathogens including *Bartonella* spp., *Anaplasma* spp. and *Rickettsia* spp., all known to be harmful to humans. Thus, we were interested to analyze and identify the microbial composition of blood-fed ticks from wild animals and pets sampled throughout Hesse. For this, a *16S* rRNA gene amplicon-sequencing (V4 region) and bioinformatics analysis workflow was established. Overall, 136 ticks sampled from 97 animals were sequenced on the MiSeq Illumina platform, resulting in a minimum sequencing depth of 5000 reads per sample for further analysis. After performing the initial NGS sequencing of 136 ticks we continued tick collection, resulting in the fact that not all of the total collected ticks (*n* = 189) underwent microbiome analysis.

**Fig. 5:**
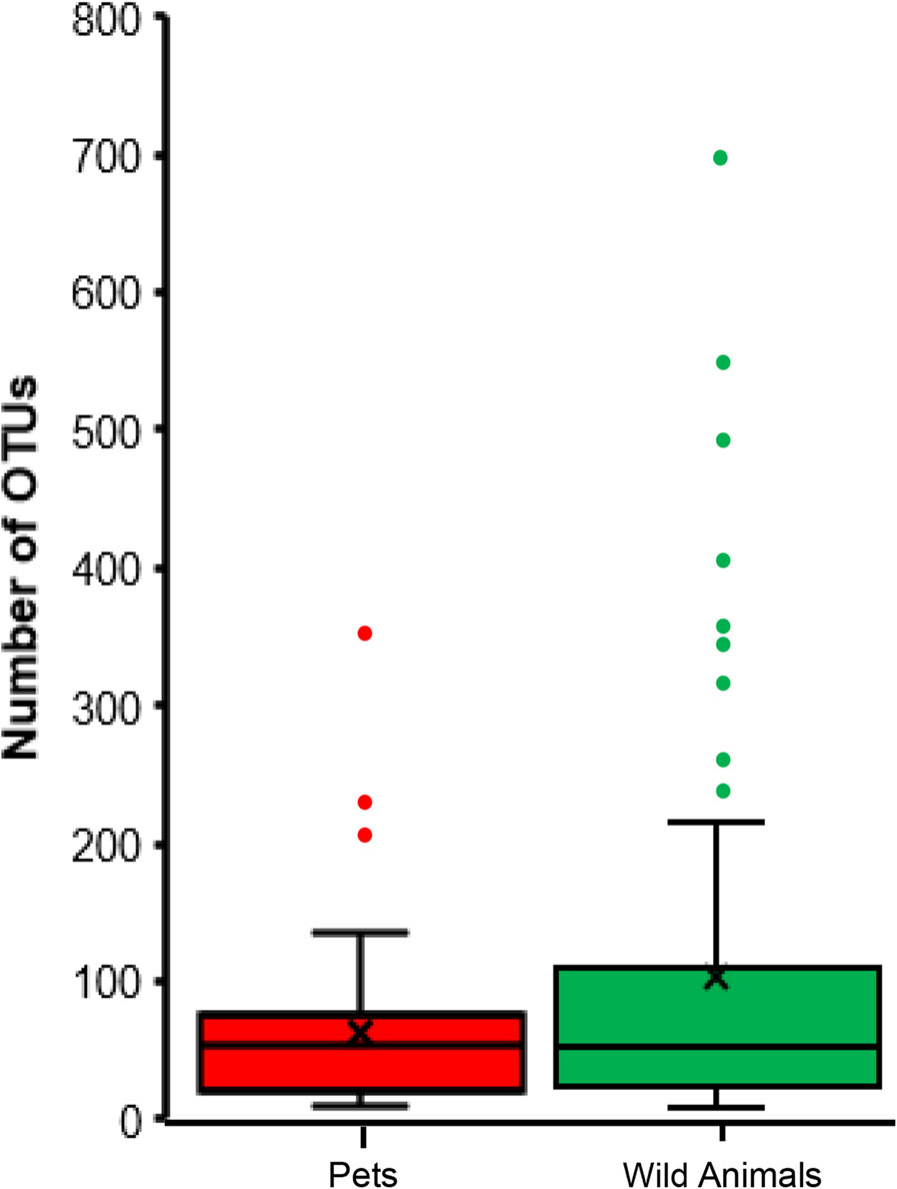
Number of operational taxonomic units (OTUs) in ticks from pets and wild animals at a sampling depth of 5000 reads. Subsampling without replacement was repeated 1000 times and averages reported

The alpha diversity of ticks sampled from wild animals showed a dominant higher number of operational taxonomic units (OTUs) compared to ticks collected from pets, indicating greater species richness in wild animals (Fig. 5). Here, we also observed more outliers and a wider range for the group of wild animals compared to the pet group.

**Fig. 6:**
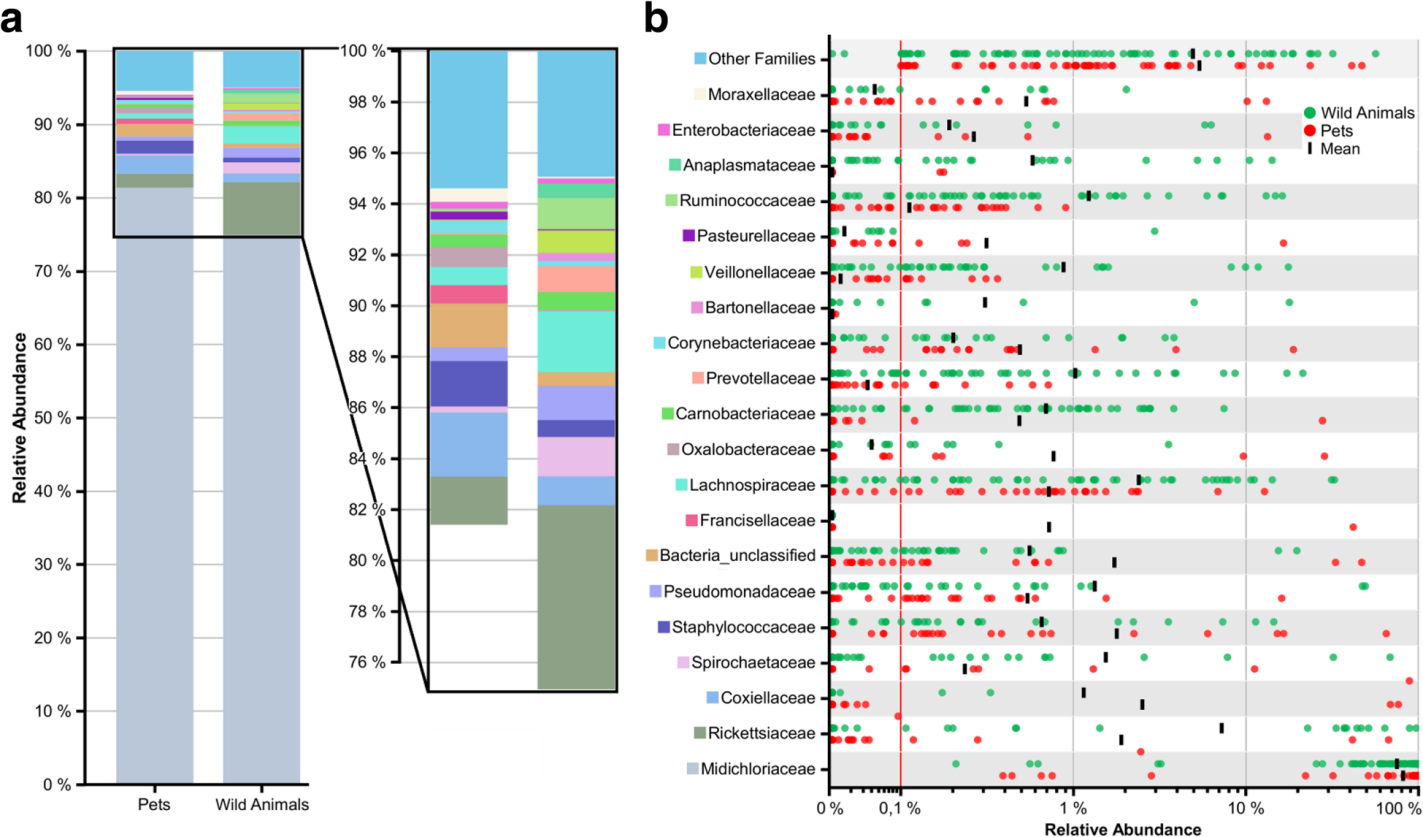
Overview of top 20 bacterial families found in ticks by NGS. **a** Cumulative bar charts comparing relative family abundances for ticks collected from pets and wild animals. **b** Variation in relative abundance of each family in tick samples. Red line shows cut-off for noise. Families not in the top 20 by relative abundance are categorized as other families

To examine the microbial taxonomic distribution of sampled ticks, cumulative bar charts composed on family level were created. Comparing both groups, we observed that *Midichloriaceae*, a common tick endosymbiont, is represented as the most dominant OTU for pets (~ 80%) and wild animals (~75%) (Fig. 6a). As seen in Fig. 6b, which represents the variation in relative abundances of the top 20 OTUs between wild animals and pets, the group of wild animals revealed a higher abundance of *Rickettsiaceae*, *Spirochaetaceae*, *Bartonellaceae* and *Anaplasmataceae*. Other tick-associated OTUs which were found more dominantly represented in ticks sampled from pets included *Coxiellaceae* and *Francisellaceae*. Interestingly, ticks of wild animals exhibit a “companion microbiome” as observed in higher abundances of *Ruminococcaceae*, *Carnobacteriaceae* and *Lachnospiraceae*, which regularly colonize the intestine of animals and can be detected in microbiome studies. We also observed the families of *Prevotellaceae* and *Veillonellaceae* enriched in wild animals, which are representatives of anaerobic bacteria known to colonize, for example, the oral cavity. A well-known issue of the microbiome analysis is the “kitome” contamination problem, which was extensively reported by several groups [58, 59]. As identification of false positive OTUs generated from DNA kits as well as PCR contaminations are critical issues in the analysis of microbiome data, kit and water controls together with the tick microbiome samples were performed. Here, we identified species of the genera *Halomonas* and *Shewanella* as potential kit DNA contaminants which were, however, absent in the top 20 families found in the herein described tick sample microbiome results. These species with low abundance were present in few samples but had no deeper impact in our further analysis. We also identified barcode crosstalk as background noise in barcode controls, but this sequencing-specific artifact did not affect the results of this study.

In order to identify bacteria to the species level, full length sequencing of the *16S* rRNA gene using Oxford Nanopore Technology was applied. Three samples revealing a higher percentage of unclassified bacteria (relative abundance > 15%) were selected in addition to two samples indicating the presence of the genus *Borrelia*. Our results revealed that the previously identified OTU “Bacteria unclassified” was resolved to *Spiroplasma ixodetis*, another known tick endosymbiont [60]. *Borrelia miyamotoi* was found in the selected two tick samples formerly by Illumina sequencing and PCR confirmation, and was also identified by Nanopore sequencing to species level.

Confirmation PCRs were conducted for all pathogenic species (Table 2). DNA of *Bartonella* spp. was detected in four ticks (5.3%) from three roe deer. Three were confirmed to be *B. schoenbuchensis* by PCR. DNA of *Spirochaetaceae* was found in 13 ticks (17.1%) from six roe deer, four dogs and one boar. The presence of *Borrelia garinii* was confirmed in one tick from a dog and that of *Borrelia miyamotoi* in one tick from another dog and two ticks from two roe deer. *Rickettsia* DNA was detected in 15 ticks (19.7%) from 10 roe deer, one raccoon, two dogs and one boar. The presence of *Rickettsia helvetica* was confirmed in nine of those ticks. *Coxiella* DNA was detected in three ticks (3.9%) from two dogs and one raccoon. Confirmation PCRs for *Coxiella burnetii* remained negative so, potentially, those *Coxiella* spp. detected by NGS and not confirmed in *Coxiella burnetii*-specific PCRs represent *Coxiella-*like tick endosymbionts [61]. *Francisella tularensis* DNA was detected in one female adult *Rhipicephalus sanguineus* tick from a dog. This result was confirmed by real-time PCR. *Anaplasma* DNA was found in 15 ticks (19.7%) from nine roe deer, one dog and one boar and *Anaplasma phagocytophilum* was confirmed to be present in 12 of those ticks. *Staphylococcus* DNA was detected in eight ticks (10.5%) from five dogs, two boars and one raccoon. Table 3 shows a summary of pathogens found by NGS data analysis.

**Table 3:** Potentially pathogenic genera found in the microbiome of 76 ticks obtained from 48 animals

OTU (*n*)	PCR confirmation (*n*)	Host species (*n*)
*Spirochaetaceae* (*n* = 13)	*B. garinii* (*n* = 1); *B. miyamotoi* (*n* = 3)	Roe deer (*n* = 6); dog (*n* = 4); boar (*n* = 1)
*Bartonella* spp. (*n* = 4)	*B. schoenbuchensis* (*n* = 3)	Roe deer (*n* = 3)
*Rickettsia* spp. (*n* = 15)	*R. helvetica* (*n* = 9)	Roe deer (*n* = 10); raccoon (*n* = 1); dog (*n* = 2); boar (*n* = 1)
*Coxiella* spp. (*n* = 3)	*C. burnetii* found	Dog (*n* = 2), raccoon (*n* = 1)
*Francisella* spp. (*n* = 1)	*F. tularensis* (*n* = 1)	Dog (*n* = 1)
*Anaplasma* spp. (*n* = 15)	*A. phagocytophilum* (*n* = 12)	Roe deer (*n* = 10); dog (*n* = 1); boar (*n* = 1)
*Staphylococcus* spp. (*n* = 8)	Not conducted	Dog (*n* = 5); boar (*n* = 2); raccoon (*n* = 1)

Whenever potentially pathogenic genera were found in the microbiome analysis, animal whole-blood (when available) was also analyzed by PCR. Here, *Anaplasma phagocytophilum* was detected in the blood of eight roe deer.

Furthermore, co-infections of ticks with various bacteria were also detected. NGS revealed that nine ticks (11.8%) were co-infected with more than one pathogen (Table 4). One tick from a boar contained DNA from *Spirochaetaceae*, *Anaplasma* spp. and *Staphylococcus* spp. In another tick from a raccoon, *Rickettsia* spp., *Coxiella* spp. and *Staphylococcus* spp. DNA were found. *Spirochaetaceae*, *Bartonella* spp. and *Anaplasma* spp. were detected in three ticks from three roe deer. *Spirochaetaceae*, *Rickettsia* spp. and *Anaplasma* spp. were found in one tick from a roe deer. Furthermore, we detected three ticks from three roe deer with co-infections with two pathogens: one tick was co-infected with *Bartonella* spp. and *Anaplasma* spp. DNA, another tick was co-infected with *Spirochaetaceae* and *Anaplasma* spp. and the third tick was co-infected with *Rickettsia* spp. and *Anaplasma* spp. DNA.

**Table 4:** Co-infections with various pathogens found in ticks taken from wild animals

Host species	*Spirochaetaceae*	*Anaplasma* spp.	*Staphylococcus* spp.	*Rickettsia* spp.	*Coxiella* spp.	*Bartonella* spp.
Boar	+	+	+	-	-	-
Raccoon	-	-	+	+	+	-
Roe deer	+	+	-	-	-	+
Roe deer	+	+	-	-	-	+
Roe deer	+	+	-	-	-	+
Roe deer	+	+	-	+	-	-
Roe deer	-	+	-	-	-	+
Roe deer	+	+	-	-	-	-
Roe deer	-	+	-	+	-	-

*Key*: + positive; - negative

### Indirect immunofluorescence assay (IIFA) of pets and pet owners

One of 54 dogs (1.9%) was seropositive for anti-*B. henselae*-antibodies (IgG) (titer: 1:640); in the respective tick, no *B. henselae* DNA was detected. One dog serum was found to be positive for *Rickettsia conorii*-antibodies (titer: 1:128); in the respective tick, *Rickettsia helvetica* DNA was detected. For all other dogs, antibodies against *Anaplasma phagocytophilum*, *Borrelia* spp., *Leptospira* spp., *Coxiella burnetii*, *Francisella tularensis* and *Rickettsia rickettsii* were not detected*.*

In five dog owners, anti-*B. henselae*-antibodies (IgG) were found (titers: 4 × 1:160, 1 × 1:320). All of the respective dogs, however, were seronegative, and, moreover, the remaining six dog owners did not show antibodies against *B. henselae*. No antibodies against *Anaplasma phagocytophilum*, *Rickettsia typhi*, *Rickettsia rickettsii*, *Borrelia burgdorferi*, *Francisella tularensis*, *Coxiella burnetii* or *Leptospira* spp. were found in the sera of the dog owners whose dogs were infested with ticks harboring a particular pathogen’s DNA.

## Discussion

Ticks and tick-borne diseases remain a remarkable health threat for humans and animals in modern days. In this study, seven zoonotic potentially human pathogenic genera (*Bartonella* spp., *Spirochaetaceae*, *Anaplasma* spp., *Rickettsia* spp., *Coxiella* spp., *Francisella* spp. and *Staphylococcus* spp.) were found in ticks feeding on pets and wild animals in Hesse, Germany, and identified using NGS and classical PCR diagnostics for microbiome profiling.

By conducting a literature search including 19 published studies, *Bartonella* spp. DNA was formerly detected in about 15% of ticks (reviewed in [62]). In the present study, *Bartonella* DNA was found to be present in ~10% of all ticks (*n* = 18 of 189 ticks in total) suggesting a similar prevalence of *Bartonella* spp. in ticks collected at different locations in the state of Hesse, Germany. DNA of the ruminant-associated *B. schoenbuchensis* and *B. capreoli* was detected in 7.4% and 0.5% of the ticks collected from roe deer, respectively. Furthermore, DNA of *B. schoenbuchensis* (25.6%), *B. capreoli* (10.3%) and DNA from both *Bartonella* spp. (7.7%) were found in the blood of roe deer. In ticks collected from nine of those *Bartonella-*positive animals, no *Bartonella* spp. was detectable indicating that these negative ticks cleared the infection or that there were not enough pathogens in the ticks to be detected by the applied PCRs. The deer ked (*Lipoptena cervi*) is suspected to be the main vector for *B. schoenbuchensis* [22, 23, 30, 63–65] and represents a common ectoparasite of roe deer and other cervids [2] which serve as reservoir hosts for the ruminant-associated *B. schoenbuchensis*, *B. capreoli*, *B. chomelii* and *B. bovis* [22–29, 66]. It remains unclear to which extent these *Bartonella* spp. can cause diseases in their reservoir or accidental human hosts but, in general, *Bartonella* spp. are known to cause chronic asymptomatic infections in their mammalian reservoir hosts [30, 31]. *Bartonella schoenbuchensis* is suspected to cause unspecific symptoms like muscle pain and fever in humans as it was isolated from one patient with a history of tick bites in which no other causative agent was detected [32]. *Bartonella* DNA was detected in ticks collected from three animals whose blood samples were free from *Bartonella* DNA, indicating that the infection might have been acquired elsewhere. This result is in-line with another study where *B. henselae* DNA-positive and negative ticks were removed from dogs at the same time, with this observation leading to the hypothesis that the DNA-positive ticks had already acquired the *B. henselae* infection before feeding on these dogs [67].

Several studies suggest that vertical transmission of *Bartonella* spp. in ticks can occur so that even larvae can possibly transmit these pathogens. *Bartonella* DNA was detected in unfed adult *I. persulcatus* [68], in unengorged larvae and nymphs of *Dermacentor variabilis* and *I. scapularis* ticks [8], in unfed *I. ricinus* adults and nymphs [69] and in questing *I. ricinus* ticks collected from France [5], indicating that these ticks acquired the pathogen by vertical transmission. Further hints arguing for ticks as vectors for *Bartonella* spp. are co-infections of animals with multiple tick-borne pathogens. Chomel et al. [70] reported, that a dog suffering from *B. clarridgeiae* endocarditis was not only *Bartonella* seropositive but also seropositive for *Anaplasma phagocytophila* which is usually transmitted by ticks [70]. Multiple co-infections with tick-borne pathogens such as *Ehrlichia* spp., *Babesia canis*, *Bartonella vinsonii* and *Rickettsia rickettsii* were found in dogs which were heavily exposed to ticks [71]. Furthermore, dogs suffering from endocarditis caused by *Bartonella* spp. were found to have high antibody titers against several tick-borne pathogens (*Anaplasma phagocytophilum*, *Rickettsia rickettsii*, *Ehrlichia canis* and *Borrelia burgdorferi* [72]). However, the fact that *B. henselae* was only detected in three ticks, leads to the conclusion that there is a low risk of acquiring *B. henselae* infections by tick bites, at least in the herein sampled area.

The microbiome analysis presented here reveals that, besides *Bartonella* spp., six other potentially pathogenic genera were detected by NGS. *Spirochaetaceae* (17.1%), *Rickettsia* spp. (19.7%), *Coxiella* spp. (3.9%), *Francisella* spp. (1.3%), *Anaplasma* spp. (19.7%) and *Staphylococcus* spp. (10.5%) were also identified in ticks. These results coincide with several studies investigating the microbiome of ticks worldwide. *Francisella* spp., *Coxiella* spp., *Rickettsia* spp. and *Shigella* spp. were detected in two tick species collected from humans in Turkey [6] and *Coxiella* spp., *Rickettsia* spp., *Anaplasma* spp., *Ehrlichia* spp., *Wolbachia* spp., *Mycobacteria* spp., *Pseudomonas* spp., *Staphylococcus* spp., *Acinetobacter* spp., *Klebsiella* spp. and *Leptospira* spp. were found in ticks removed from domestic animals in Malaysia [7]. In Indiana, USA, *Francisella* spp., *Rickettsia* spp. and *Bartonella* spp. were detected in ticks removed from small rodents [8], *Anaplasma* spp., *Borrelia* spp., *Coxiella* spp., *Ehrlichia* spp., *Francisella* spp. and *Rickettsia* spp. were found in questing ticks collected by flagging in France [9] and ticks collected from dogs and the environment in France, Senegal and USA showed presence of *Rickettsia* spp., *Coxiella* spp. and *Bacillus* spp. [11]. All these pathogens are known to be transmitted by ticks, occur worldwide and can cause infections in humans. Our results suggest that there is no huge difference between the tick microbiome in warmer regions e.g. Turkey and Malaysia [6, 7] and our sampling sites in Hesse, Germany.

Confirmatory PCRs conducted depending on the NGS results revealed the presence of *Borrelia garinii*, *Borrelia miyamotoi*, *Rickettsia helvetica*, *Francisella tularensis* and *Anaplasma phagocytophilum*; these results were widely congruent with those from the NGS approach. All of these bacterial species can cause unspecific febrile illnesses in humans and probably also in pets [73–81]. Furthermore, *Anaplasma phagocytophilum* was detected in the blood of eight roe deer. Compared to the average prevalence of *Borrelia burgdorferi* (*sensu lato*) in questing ticks in Europe (12.3% [82]), the DNA-prevalence in this study is quite low. A possible explanation for this phenomenon could be that, in contrast to most other studies, we examined feeding ticks from different vertebrate species and it has been shown that the complement system of several vertebrates, especially ruminants, can effectively eliminate Lyme disease spirochetes inside the feeding ticks [83, 84].

Nine ticks showed co-infections with two or more pathogens. Co-infections of ticks with different *Borrelia* spp. or *Borrelia* spp. and other pathogens such as *Anaplasma phagocytophilum* or *Rickettsia* spp. are not rare [85] and they can alter the course of human infection or the response to a certain antimicrobial therapy. Patients with chronic, therapy-resistant Lyme disease showed co-infections with *B. henselae* leading to the assumption that infections with multiple microorganisms might cause irregular responses to antibiotic therapies [40]. From this, knowledge about co-infections of ticks with various pathogens, even when limited to a relatively small area as in our study, is mandatory to guide for best antimicrobial treatment of patients after tick exposure.

A limitation of our study is the fact that blood samples were taken at the same time point as tick collection was performed. From this, pathogens detected in those ticks might not have induced an actual serological response in infected hosts as ticks feed on dogs normally for only 2–10 days [2]. Furthermore, since we decided to work on feeding ticks, there are two possibilities by nature where the detected bacteria might derive from: (i) from the tick, which can possibly act as a vector and transmit those pathogens to different vertebrates; or (ii) the pathogens were already present in the blood of the host animal and were ingested while feeding.

Anti-*Bartonella* antibodies can be found in up to 16.1% of healthy blood donors [86]. In five of 11 dog owners investigated herein, anti-*B. henselae*-IgG antibodies were detected with titers between 1:160 to 1:320, resulting in an antibody prevalence of ~45%. Such relatively low titers might be an indicator for a former infection by *Bartonella* spp. and the percentage of *B. henselae*-positive dog owners seems to be elevated. However, it has to be mentioned that this elevated seroprevalence might also be caused by exposure to other *Bartonella* spp. as exact data on cross-reactivity of human anti-*Bartonella* antibodies with *B. henselae* antigen (which is used for serodiagnostics) are not available.

## Conclusions

In summary, our data provide an overview of different pathogens circulating in ticks in central Germany (federal state of Hesse) and suggest a low incidence of *Bartonella* spp. in animals and their ectoparasites. Members of several pathogenic genera were found in ticks and also in wild and domestic animals by PCR and NGS techniques with Illumina short read and Nanopore long read sequencing, and this indicates a potential infection risk for humans and animals. Even though the number of samples used herein was too small to evaluate the epidemiology of different tick-borne diseases, knowledge of the presence of pathogens in ticks might allow to monitor circulating pathogens that could harm humans and animals. Ectoparasite control and an increased attention toward possible tick-borne infection are crucial to the prevention of (or at least early diagnosis of) tick-borne infections in humans and animals.

## Abbreviations

NGS: Next generation sequencing
rDNA: Ribosomal DNA
ITS: Internal transcribed spacer
OTU: Operational taxonomic unit
